# Supplementary material for: Coordinated head direction representations in mouse anterodorsal thalamic nucleus and retrosplenial cortex
Source: eLife. 2024 Mar 12;13:e82952. doi: 10.7554/eLife.82952 (PMC10932540; doi:10.7554/eLife.82952)
Supplement: MDAR checklist [file elife-82952-mdarchecklist1.docx]

**Materials Design Analysis Reporting (MDAR)**

**Checklist for Authors**

The [MDAR framework](https://osf.io/xfpn4/) establishes a minimum set of requirements in transparent reporting mainly applicable to studies in the life sciences.

*eLife* asks authors to **provide detailed information within their article** to facilitate the interpretation and replication of their work. Authors can also upload supporting materials to comply with relevant reporting guidelines for health-related research (see [EQUATOR Network](http://www.equator-network.org/%20)), life science research (see the [BioSharing Information Resource](http://biosharing.org/)), or animal research (see the [ARRIVE Guidelines](http://www.plosbiology.org/article/info:doi/10.1371/journal.pbio.1000412) and the [STRANGE Framework](https://doi.org/10.1038/d41586-020-01751-5); for details, see *eLife*’s [Journal Policies](https://reviewer.elifesciences.org/author-guide/journal-policies)). Where applicable, authors should refer to any relevant reporting standards materials in this form.

For all that apply, please note **where in the article** the information is provided. Please note that we also collect information about data availability and ethics in the submission form.

**Materials:**

| **Newly created materials** | **Indicate where provided: section/figure legend** | **N/A** |
| --- | --- | --- |
| The manuscript includes a dedicated "materials availability statement" providing transparent disclosure about availability of newly created materials including details on how materials can be accessed and describing any restrictions on access. |  | N/A |
|  |  |  |
| **Antibodies** | **Indicate where provided: section/figure legend** | **N/A** |
| For commercial reagents, provide supplier name, catalogue number and [RRID](https://scicrunch.org/resources), if available. | DAPI, 62248; Thermo Fisher Scientific; Fig. 1 – Supplement 1A, Fig. 4A,B, Fig. 4 – Supplement 2 |  |
|  |  |  |
| **DNA and RNA sequences** | **Indicate where provided: section/figure legend** | **N/A** |
| Short novel DNA or RNA including primers, probes: Sequences should be included or deposited in a public repository. |  | N/A |
|  |  |  |
| **Cell materials** | **Indicate where provided: section/figure legend** | **N/A** |
| Cell lines: Provide species information, strain. Provide accession number in repository OR supplier name, catalog number, clone number, OR RRID. |  | N/A |
| Primary cultures: Provide species, strain, sex of origin, genetic modification status. |  | N/A |
|  |  |  |
| **Experimental animals** | **Indicate where provided: section/figure legend** | **N/A** |
| Laboratory animals or Model organisms: Provide species, strain, sex, age, genetic modification status. Provide accession number in repository OR supplier name, catalog number, clone number, OR RRID. | 4 females and 9 males mice C57BL/6 from Charles River and from Jackson Laboratory (RRID: IMSR_JAX:000664) and one male mouse Vgat-Ires-Cre C57 BL/6 (RRID:IMSR_RBRC10723). All mice employed for recordings (12) were 8-11 weeks old at the time of surgery and behavior recordings followed in the next 3-6 months. The 4 mice employed for anatomical tracing were 12 weeks old at the time of surgery. |  |
| Animal observed in or captured from the field: Provide species, sex, and age where possible. |  | N/A |
|  |  |  |
| **Plants and microbes** | **Indicate where provided: section/figure legend** | **N/A** |
| Plants: provide species and strain, ecotype and cultivar where relevant, unique accession number if available, and source (including location for collected wild specimens). |  | N/A |
| Microbes: provide species and strain, unique accession number if available, and source. |  | N/A |
|  |  |  |
| **Human research participants** | **Indicate where provided: section/figure legend) or state if these demographics were not collected** | **N/A** |
| If collected and within the bounds of privacy constraints report on age, sex, gender and ethnicity for all study participants. |  | **N/A** |

**Design:**

| **Study protocol** | **Indicate where provided: section/figure legend** | **N/A** |
| --- | --- | --- |
| If the study protocol has been pre-registered, provide DOI. For clinical trials, provide the trial registration number OR cite DOI. |  | N/A |
|  |  |  |
| **Laboratory protocol** | **Indicate where provided: section/figure legend** | **N/A** |
| Provide DOI OR other citation details if detailed step-by-step protocols are available. |  | N/A |
|  |  |  |
| **Experimental study design (statistics details) *** | | |
| **For in vivo studies: State whether and how the following have been done** | **Indicate where provided: section/figure legend. If it could have been done, but was not, write “not done”** | **N/A** |
| Sample size determination | Mouse number sample size was not precomputed, but rather determined based on experience in the field and comparison with similar studies. Number of sessions per mouse varied from 2 to 15 depending on the quality of the recordings and sampling of HD units. |  |
| Randomisation |  | **N/A** |
| Blinding |  | N/A |
| Inclusion/exclusion criteria | Mice from which we did not record at least 2 HD thalamic cells were not included in this study. Sessions that did not contain cue manipulations were not included in the analysis, and trials or sessions that had poor spatial occupancy were excluded. Sorted units that did not |  |
|  |  |  |
| **Sample definition and in-laboratory replication** | **Indicate where provided: section/figure legend** | **N/A** |
| State number of times the experiment was replicated in the laboratory. | 12 mice were used for recordings, of which 9 had tetrodes implanted simultaneously in ADn and RSC, 1 had carbon fibers implanted only in ADn and 2 mice had tetrodes only in RSC. Mice underwent several recording sessions (between 2 and 15) and each session contained 1 to 11 trials. 4 mice were used for anatomical tracing. |  |
| Define whether data describe technical or biological replicates. | Data was analyzed by first determining which recorded neuronal ensembles were not overlapping and, for some analyses, averaging across similar neuronal ensembles before combining them in the final plots and performing statistical tests, while for others selecting only the independent ones. Therefore, replicates are both biological and technical. |  |
|  |  |  |
| **Ethics** | **Indicate where provided: section/submission form** | **N/A** |
| Studies involving human participants: State details of authority granting ethics approval (IRB or equivalent committee(s), provide reference number for approval. |  | N/A |
| Studies involving experimental animals: State details of authority granting ethics approval (IRB or equivalent committee(s), provide reference number for approval. | Massachusetts  Institute of Technology Committee on Animal care, protocol number 0521-036-24 |  |
| Studies involving specimen and field samples: State if relevant permits obtained, provide details of authority approving study; if none were required, explain why. |  | N/A |
|  |  |  |
| **Dual Use Research of Concern (DURC)** | **Indicate where provided: section/submission form** | **N/A** |
| If study is subject to dual use research of concern regulations, state the authority granting approval and reference number for the regulatory approval. |  | N/A |

**Analysis:**

| **Attrition** | **Indicate where provided: section/figure legend** | **N/A** |
| --- | --- | --- |
| Describe whether exclusion criteria were pre-established. Report if sample or data points were omitted from analysis. If yes, report if this was due to attrition or intentional exclusion and provide justification. | For certain analyses pertaining HD unit properties (Fig. 1C,F,G, Fig. 1 - Supplement 3B-L) and functional connectivity (Fig. 4, Fig.4 – Supplement 2), quantifications were based on a subset of sessions with independent ensembles of units, based on tetrode movements and number of recorded units. Figure legends, main text and method sections indicate how trials with overlapping ensembles were averaged and considered independent samples depending on: whether thalamic and cortical units were simultaneously recorded (Fig. 2B,D&E, Fig. 2 - Supplement 2B, Fig. 3 – Supplement 1A&B) and according to the size of the rotation (Fig. 1H and Fig.1 – Supplement 3M, Fig. 2 – Supplement 1G&H); whether the mean decoded rotation value was above 17.2° (Fig. 2D&E and Fig. 2 – Supplement 3), whether a significant tuning curve rotation was observed (Fig. 1F&G). |  |
|  |  |  |
| **Statistics** | **Indicate where provided: section/figure legend** | **N/A** |
| Describe statistical tests used and justify choice of tests. | N-ways anova was used to test the effects of different grouping (Fig. 2 - Supplements 1F and 2B); otherwise Kruskal Wallis tests followed by Bonferroni multiple comparisons were used (Fig.1 - Supplement 3B&C) to test effects across multiple groups and non-parametric Wilcoxon Signed-Rank (Fig. 2E, Fig. 1 - Supplement 3G-L, Fig. 3F&G, Fig. 3 - Supplement 1A&B and Fig.2 - Supplement 3A,D,F between shifted and stable) for paired tests, and Mann-Whitney tests between two groups were used between to avoid assumptions (Fig.2 – Supplement 1D). KS-tests were used to compare the distributions between the real and shuffled time lags of peak correlation (Fig. 2E, and Fig. 2 - Supplement 3A). Rayleigh test was applied to the non-uniformity of circular data in Fig. 4G. Null distributions were generated from shuffling to compare against real effects (Fig. 1B,H, Fig. 1 - Supplement 3A, Fig. 2B&E, Fig. 2 – Supplement 1G&H, Fig. 2 - Supplement 3A&C, Fig. 3D&E). |  |
|  |  |  |
| **Data availability** | **Indicate where provided: section/submission form** | **N/A** |
| For newly created and reused datasets, the manuscript includes a data availability statement that provides details for access (or notes restrictions on access). | Data will be available on Dryad upon publication. |  |
| When newly created datasets are publicly available, provide accession number in repository OR DOI and licensing details where available. | Revised data: doi:10.5061/dryad.dfn2z3555, under creative common license CC0 1.0 Universal |  |
| If reused data is publicly available provide accession number in repository OR DOI, OR URL, OR citation. |  | N/A |
|  |  |  |
| **Code availability** | **Indicate where provided: section/figure legend** | **N/A** |
| For any computer code/software/mathematical algorithms essential for replicating the main findings of the study, whether newly generated or re-used, the manuscript includes a data availability statement that provides details for access or notes restrictions. | Associated code will be available on Dryad upon publication |  |
| Where newly generated code is publicly available, provide accession number in repository, OR DOI OR URL and licensing details where available. State any restrictions on code availability or accessibility. | Revised code doi:10.5061/dryad.dfn2z3555, under creative common license CC0 1.0 Universal |  |
| If reused code is publicly available provide accession number in repository OR DOI OR URL, OR citation. |  | N/A |

**Reporting:**

The MDAR framework recommends adoption of discipline-specific guidelines, established and endorsed through community initiatives.

| **Adherence to community standards** | **Indicate where provided: section/figure legend** | **N/A** |
| --- | --- | --- |
| State if relevant guidelines (e.g., ICMJE, MIBBI, ARRIVE, STRANGE) have been followed, and whether a checklist (e.g., CONSORT, PRISMA, ARRIVE) is provided with the manuscript. |  | N/A |

* We provide the following guidance regarding transparent reporting and statistics; we also refer authors to [Ten common statistical mistakes to watch out for when writing or reviewing a manuscript](https://doi.org/10.7554/eLife.48175).

**Sample-size estimation**

- You should state whether an appropriate sample size was computed when the study was being designed
- You should state the statistical method of sample size computation and any required assumptions
- If no explicit power analysis was used, you should describe how you decided what sample (replicate) size (number) to use

**Replicates**

- You should report how often each experiment was performed
- You should include a definition of biological versus technical replication
- The data obtained should be provided and sufficient information should be provided to indicate the number of independent biological and/or technical replicates
- If you encountered any outliers, you should describe how these were handled
- Criteria for exclusion/inclusion of data should be clearly stated
- High-throughput sequence data should be uploaded before submission, with a private link for reviewers provided (these are available from both GEO and ArrayExpress)

**Statistical reporting**

- Statistical analysis methods should be described and justified
- Raw data should be presented in figures whenever informative to do so (typically when N per group is less than 10)
- For each experiment, you should identify the statistical tests used, exact values of N, definitions of center, methods of multiple test correction, and dispersion and precision measures (e.g., mean, median, SD, SEM, confidence intervals; and, for the major substantive results, a measure of effect size (e.g., Pearson's r, Cohen's d)
- Report exact p-values wherever possible alongside the summary statistics and 95% confidence intervals. These should be reported for all key questions and not only when the p-value is less than 0.05.

**Group allocation**

- Indicate how samples were allocated into experimental groups (in the case of clinical studies, please specify allocation to treatment method); if randomization was used, please also state if restricted randomization was applied
- Indicate if masking was used during group allocation, data collection and/or data analysis
