# Supplementary material for: Coordinated head direction representations in mouse anterodorsal thalamic nucleus and retrosplenial cortex
Source: eLife. 2024 Mar 12;13:e82952. doi: 10.7554/eLife.82952 (PMC10932540; doi:10.7554/eLife.82952)
Supplement: Supplementary file 1. [file elife-82952-supp1.pdf]

Table 1: Recording sessions summary

mouse	Date	Session Type	ADn HD	ADn total	RSC HD	RSC total	ADn → RSC	RSC → ADn	Pairs total	Electrodes and advancement – fraction of turns											New ADn HD ensemble	New RSC HD ensemble	New ADn rotation ensemble	New RSC rotation ensemble	New ADn dark ensemble	New RSC dark ensemble
mouse 1	carbon	fiber			N/A	N/A	N/A	N/A	N/A																	
	4/11/19	on/off	14	19																					✓	
	4/26/19	on/off	15	23																						
	4/30/19	rotations+off	11	18																			✓			
	5/5/19	rotations+off	9	15																						
	5/29/19	on/off	8	31																						
	6/9/19	on/off	11	18																						
	6/18/19	rotations+off	15	20																			✓			
	8/9/19	rotations	9	18																						
	8/12/19	rotations	16	21																	✓					
	10/27/19	rotations	20	26																			✓			
	10/28/19	on/off	3	27																						
	10/31/19	rotations+off	23	27																						
mouse 2										RSC	RSC	RSC	RSC	RSC	N	ADn	ADn	ADn								
										16	15	14	13	1,2	10,9	8,7	5, 6	3,4								
	10/05/19	rotations+off	4	8	1	11	0	0	88					0.125	0.125		0.125				✓	✓	✓	✓	✓	
	10/08/19	rotations+off	1	8	1	9	0	0	72					0.125			0.125						✓			✓
	10/09/19	rotations	1	6	0	7	0	0	42												✓					
	10/09/19	rotations	0	5	0	7	0	0	35	0.125	0.125			0.125	0.125		0.125									
	10/10/19	rotations	2	13	0	10	0	0	130					0.375	0.125	0.25	0.375	0.125				✓	✓	✓		
	10/14/19	rotations+off	6	13	0	11	0	0	143			0.25	0.25	0.125							✓		✓		✓	
	10/18/19	rotations	5	14	0	14	0	0	210			0.5	0.5					-0.125				✓		✓		
	10/21/19	rotations+off	4	14	0	12	0	0	140					0.4	0.4	-0.1							✓			
	10/27/19	rotations+off	8	16	1	13	0	0	208												✓		✓	✓	✓	
	10/28/19	on/off	8	19	2	11	0	0	209	0.3	0.3															
	10/31/19	rotations	7	19	0	18	0	0	342						0.25	0.125		-0.1				✓		✓		
	12/03/19	rotations+off	12	19	0	14	0	0	266						0.25	0.125		0.125			✓		✓			✓
	01/07/20	rotations	8	15	0	16	0	0	225												✓		✓			
mouse 3			N/A	N/A			N/A	N/A	N/A																	
	02/22/20	rotations			6	92																		✓		
	02/23/20	rotations			14	94																				
	02/24/20	rotations			2	92																				
	02/29/20	rotations			4	104																		✓		
	03/17/20	rotations			13	104																				
	03/19/20	rotations			7	101																				
	03/20/20	rotations			4	100																				
	03/21/20	rotations			6	104																				
	03/24/20	rotations			24	96																				
	03/25/20	rotations			17	104																✓				
	03/26/20	rotations			24	107																				
	03/27/20	rotations			16	105																				
	03/29/20	rotations			14	97																		✓		
	03/31/20	rotations			15	98																				
mouse 4										RSC	RSC	RSC	RSC	RSC	ADn	N	N	N	N	ADn						
										9	10	11	12	13	14, 15	16,1	2,3	4,5	6,7	8						
	02/28/20	rotations	2	6	1	33	0	0	198								0.125		0.125	0.125	✓	✓	✓	✓		
	03/02/20	rotations	2	8	3	31	0	0	248											0.125						
	03/07/20	rotations	1	7	6	42	0	0	287						0.125	0.25	0.125	1	0.25	0.25		✓		✓		
	03/12/20	rotations	1	4	9	39	0	0	156						0.125	0.125	0.125	0.125	0.125	0.125			✓			
	03/13/20	rotations	4	6	6	35	0	0	210						0.125	0.25		0.25	0.25	0.25	✓		✓			
	03/16/20	rotations	1	6	11	39	0	0	195						0.1			-0.1		-0.75						
	03/19/20	rotations	1	4	3	31	0	0	124						-0.125	-0.125		-0.125		0.75		✓	✓	✓		
	03/20/20	rotations	1	7	4	34	0	0	238						-0.025	-0.125		-0.025	-0.25	-0.025	✓					
	03/23/20	rotations	0	5	5	34	0	0	170						-0.25			0.125		0.5			✓			
	03/25/20	rotations	2	12	9	31	0	0	372						0.1								✓			
	03/26/20	rotations	3	11	1	29	0	0	319						0.725	0.125	0.125	1.25	0.125		✓					
	03/31/20	rotations	3	11	2	33	0	0	363									-0.75								
	04/04/20	rotations	2	10	2	28	0	0	280									-0.25		0.125						
	04/05/20	rotations	5	11	3	30	0	0	300						0.25			-0.5								
mouse 5										RSC	RSC	RSC	RSC	RSC	N	N	ADn	N	N							
										15, 16	14	13, 12	2	1	11	10,9	5, 6	4	3							
	03/23/20	rotations	2	3	5	26	10	0	78												✓	✓	✓	✓		
	03/24/20	rotations	3	4	4	25	14	0	100								0.125									
	03/28/20	rotations	3	3	3	27	10	0	81		0.125	2			0.125	0.125	0.125		1							

	03/31/20	rotations	3	3	3	27	11	0	81												✓		✓			
	04/20/20	rotations	2	5	4	31	17	0	140												✓	✓	✓	✓		
	04/21/21	rotations	1	5	2	24	12	0	115		0.25	0.25			0.4	0.4	0.25		0.25					✓		
	04/24/20	rotations	1	3	2	30	9	0	87		0.375	0.375			0.375	0.375	0.125		0.375		✓	✓	✓	✓		
	04/28/20	rotations	3	3	3	23	10	0	69		0.125	0.125			0.125	0.125	0.125		0.125					✓		
	05/01/20	rotations	2	3	0	22	12	0	66																	
mouse 6										RSC	RSC	RSC			ADn	N										
										7	2	1			9,8	11, 10										
	03/23/20	rotations	4	4	0	1	0	0	4												✓		✓			
	03/24/20	rotations	4	4	1	4	0	0	16													✓				
	03/26/20	rotations	2	2	0	0	0	0	0	0.25	0.24	0.25				0.25										
	03/27/20	rotations	2	3	0	0	0	0	0						0.125											
	03/31/20	rotations	4	5	0	0	0	0	0							0.25					✓		✓			
	04/10/20	rotations	4	6	0	0	0	0	0																	
	04/11/20	rotations	3	5	0	0	0	0	0							0.25										
	04/15/20	rotations	5	9	0	0	0	0	0												✓					
	04/17/20	rotations+off	5	7	0	2	0	0	14														✓		✓	
	04/18/20	rotations	4	8	0	2	0	0	14																	
	04/19/20	rotations+off	5	7	0	1	0	0	7			0.25			0.125	-0.25										
	04/20/20	rotations	4	5	0	2	0	0	10												✓		✓			
	04/20/20	rotations+off	3	4	0	2	0	0	8																	
	04/21/20	rotations	5	7	0	3	0	0	21																	
mouse 7										RSC	RSC	RSC	RSC	N	ADn	ADn	N									
										6,7	9,8	10, 11	12, 13	14, 15	16,1	2,3	4,5									
	07/02/20	rotations	4	8	1	38	14	0	296	0.25					0.375		0.375				✓	✓	✓	✓		
	07/07/20	rotations	5	5	6	34	20	1	170												✓	✓	✓	✓		
	07/09/20	rotations	4	5	0	29	16	0	145																	
mouse 8										RSC	RSC	RSC	RSC	N	ADn	N	N									
										12, 13	14, 15	16,1	2,3	4,5	6,7	8,9	10, 11									
	07/22/20	rotations	4	9	1	19	37	0	171												✓	✓	✓	✓		
	07/24/20	rotations	3	10	0	13	21	0	130																	
mouse 9										RSC	RSC	RSC	RSC	RSC	N	N	ADn	N	ADn							
										6	7,8	9	10,1 1	12,1 3	14	15	16,1	2,3	4,5							
	01/26/21	on/off	3	4	3	20	19	0	80	-0.25					0.5		-0.75		0.125		✓	✓			✓	✓
	02/04/21	on/off	1	3	4	12	6	0	36	0.125					0.125		0.125				✓				✓	✓
	02/06/21	on/off	2	4	2	17	11	0	68	0.25							-1	-0.25								
	02/11/21	on/off	2	3	3	21	11	0	63																✓	
	02/12/21	rotations	2	4	2	19	22	0	76														✓	✓		
	02/12/21	rotations	2	4	5	20	32	0	80						0.25		0.125									
	02/19/21	on/off	2	4	1	19	13	2	76	0.25	0.25		0.125		0.125		0.25	0.25				✓				
	03/07/21	rotations	2	4	0	14	15	0	56		0.25	1	0.125	0.125			0.125	0.125	0.125			✓		✓		
	03/12/21	on/off	4	5	5	13	24	0	65												✓	✓			✓	✓
	03/15/21	rotations	3	5	3	15	23	0	75														✓	✓		
	03/17/21	rotations	2	4	4	15	20	0	60	-1	-1	1	0.25	0.125			0.125	0.125	0.25							
	03/20/21	rotations	3	9	4	17	19	1	153	2	1.5	0.5	0.125	0.125				-0.5	0.25		✓		✓	✓		
	03/23/21	on/off	4	10	5	13	14	0	130																✓	✓
	06/24/21	on/off	3	4	5	20	25	0	80													✓			✓	✓
	06/25/21	on/off	2	4	2	16	19	0	64																	
	06/26/21	on/off	2	3	2	15	14	0	45	0.25		0.25														✓
	06/27/21	on/off	2	3	2	14	13	0	42																	
mouse 10										RSC	RSC	RSC	RSC	RSC	N	N	N	N	N							
			N/A	N/A			N/A	N/A	N/A	9	8	7	6,5	4	2,3	1,16	14, 15	12, 13	11, 10							
	03/18/20	rotations			0	18											0.25	0.25	0.25			✓		✓		
	03/20/23	rotations			4	22																				
	03/23/20	rotations			2	18																				
	03/24/20	rotations			1	13																		✓		
	03/26/20	rotations			0	24									0.125	0.125	0.125	0.125	0.125			✓		✓		
	04/20/20	rotations			1	17																		✓		
mouse 11										RSC	RSC	RSC	RSC	RSC	N	N	N	ADn	ADn							
										11	10,9	8	6,7	5	2,3	1,16	15, 14	13	12							
	03/02/20	rotations	2	6	7	30	18	0	180	0.25	0.25		-0.125		-0.25	-0.125	1.825	1			✓	✓	✓	✓		
	03/27/20	rotations	0	5	2	33	13	0	160												✓	✓	✓	✓		
mouse 12										RSC	RSC	RSC	RSC	RSC	ADn /AV	ADn	N	N								
										6,7	8	9,10	12, 11	13	16, 15	1,2	3,4	5								
	01/15/21	rotations	3	5	1	8	13	0	40	0.375		0.25	0.375	0.5	0.5	0.625	0.625	0.125			✓	✓	✓	✓		
	01/19/21	rotations	5	8	1	6	14	0	42	0.2		0.2	0.2				0.125	0.125			✓		✓	✓		
	01/21/21	on/off	3	6	3	6	16	0	36	-1.975		0.875	0.625		0.325	0.5	1.375	0.625							✓	✓
	02/04/21	on/off	4	7	1	8	14	0	56	3					0.125	0.125	0.125				✓				✓	✓

	02/10/21	on/off	4	8	1	7	10	0	56	1.5	0.5				0.625	-0.5	-0.325	0.25								
	03/05/21	rotations	6	13	0	6	10	0	78							0.125	0.125				✓	✓	✓	✓		
	03/11/21	on/off	7	11	0	5	3	0	55																✓	
	03/11/21	on/off	3	9	1	5	2	0	45		0.125				0.125	0.25	0.125									✓
	03/13/21	on/off	6	10	0	7	9	0	70							0.25	0.125	0.25			✓				✓	
	03/17/21	rotations	6	10	1	7	13	0	70		0.25			0.125	0.25	0.25	0.5						✓			
	03/20/21	rotations	4	10	0	7	15	0	70													✓		✓		
	03/22/21	on/off	5	10	0	7	9	0	70					0.125	0.125	0.5	0.5	0.125								
	06/25/21	on/off	7	13	1	6	3	0	78												✓				✓	
	06/26/21	on/off	6	13	2	7	3	0	91																	
	06/27/21	on/off	6	12	1	5	3	0	60																	✓
